# Prevalence of Dental Pain in Schoolchildren Aged 6 to 12 Years and Clinical, Sociodemographic, and Socioeconomic Risk Indicators: A Multicenter Study of Four Mexican Cities

**DOI:** 10.3390/pediatric16040089

**Published:** 2024-11-22

**Authors:** Manuel Jesús Godínez-López, Martha Mendoza-Rodríguez, María de Lourdes Márquez-Corona, Sandra Isabel Jiménez-Gayosso, Mauricio Escoffié-Ramírez, Nuria Patiño-Marín, Juan José Villalobos-Rodelo, Juan Fernando Casanova-Rosado, Alejandro José Casanova-Rosado, Carlo Eduardo Medina-Solís

**Affiliations:** 1 Master’s Program in Biomedical and Health Sciences of the Academic Area of Medicine, Autonomous University of the State of Hidalgo, Pachuca 42130, Mexico; mgodinezl001@uaemex.mx; 2 Academic Area of Dentistry, Health Sciences Institute, Autonomous University of Hidalgo State, Pachuca 42130, Mexico; lmarquez@uaeh.edu.mx (M.d.L.M.-C.); sandra_jimenez@uaeh.edu.mx (S.I.J.-G.); 3 School of Dentistry, Autonomous University of Yucatan, Mérida 97000, Mexico; mauricio.escoffie@correo.uady.mx; 4 Clinical Research Laboratory, School of Stomatology, Autonomous University of San Luis Potosi, San Luis Potosi 78290, Mexico; nuriapaty@uaslp.mx; 5 School of Dentistry, Autonomous University of Sinaloa, Culiacan 80040, Mexico; juanvillalobos@uas.edu.mx; 6 School of Dentistry, Autonomous University of Campeche, Campeche 24039, Mexico; jfcasano@uacam.mx (J.F.C.-R.); ajcasano@uacam.mx (A.J.C.-R.); 7 Advanced Studies and Research Centre in Dentistry “Dr. Keisaburo Miyata”, School of Dentistry, Autonomous University of the State of Mexico, Toluca 50130, Mexico

**Keywords:** oral health, dental pain, schoolchildren, Mexico

## Abstract

**Background:** Dental pain is a significant public health issue globally and in Mexico, with substantial negative impacts on affected individuals. **Objective:** The objective of this study was to evaluate the prevalence of dental pain and its association with clinical, sociodemographic, and socioeconomic risk indicators in schoolchildren aged 6 to 12 years in four cities in Mexico. **Material and Methods:** A cross-sectional study was conducted on 500 children attending public schools in the cities of Pachuca, Tepatitlán, Toluca, and San Luis Potosí. A questionnaire was applied, and a clinical examination was performed on the schoolchildren. Self-reported dental pain within the 12 months preceding the study, categorized as 0 (no pain) and 1 (pain), was used as the dependent variable. For the statistical analysis, the Stata program was used, where a multivariate binary logistic regression model was applied. **Results:** The mean age was 8.92 ± 1.99 years; 50% were girls. The prevalence of dental pain was 34.0%. Independent variables associated (*p* < 0.05) with the experience of dental pain were as follows: age (OR = 0.81), having a car at home (OR = 0.77), a lower socioeconomic position (OR = 2.03), curative or specialized dental care (OR = 8.40), and self-reported dental and/or gingival disease (OR = 5.39). **Conclusions:** Dental pain is a significant health problem in schoolchildren aged 6 to 12 years in Mexico, with a prevalence of 34% in this study. Sociodemographic and socioeconomic factors, as well as clinical indicators, play an important role in the experience of dental pain. Inequalities in oral health were observed. There is a need for public health interventions to reduce this problem in vulnerable populations.

## 1. Introduction

According to the World Health Organization’s global report on oral health, oral diseases affect approximately 3.5 billion people, with three-quarters of them living in middle-income countries. It is estimated that 2 billion people have cavities in permanent teeth, and 514 million children suffer from caries in primary teeth. These diseases disproportionately impact people in lower socioeconomic positions. There is a clear relationship between socioeconomic status (income, employment, and education) and the prevalence and severity of these diseases, from childhood to old age, across all social groups and countries. Therefore, oral diseases represent a global health problem and a challenge for health systems [1]. Oral health serves multiple functions, including speech, smiling, taste, chewing, swallowing, and emotional expression. It refers to the absence of pain, discomfort, and disease in the craniofacial complex [2]. Oral health plays a crucial role in the physical, mental, social, and economic well-being of individuals and populations. The oral cavity and each of the surrounding structures are vital to the human body and essential for daily performance, also contributing significantly to the overall well-being of an individual [3]. One of the most common problems resulting from oral diseases is toothache, which not only affects people’s quality of life but also interferes with daily activities such as eating, speaking, and sleeping [4]. This type of pain is an alarming symptom that can result from untreated cavities, infections, or gum problems, and is especially prevalent in low-income communities, where access to preventive and curative dental treatments is limited [5]. Toothache impacts not only physical health but also emotional well-being and the ability to concentrate, particularly in children, where it can lead to school absenteeism and learning difficulties [6].

The International Association for the Study of Pain (IASP) defines pain as “an unpleasant sensory and emotional experience associated with, or resembling that associated with, actual or potential tissue damage”, and this is expanded with the addition of six key notes and the etymology of the word pain for more valuable context [7,8]. Meanwhile, dental pain (toothache, odontogenic pain) refers to “pain that originates in the teeth or their supporting structures, the mucosa, gums, maxilla, jaw, or periodontal membrane” [9]. Toothache is identified as a common symptom of oral diseases such as dental caries, abscesses, and dentoalveolar trauma. However, it is also related to tooth eruption, the exfoliation of primary teeth, and gingivitis, which are capable of causing toothache. It is one of the main reasons people seek treatment, having a significant impact on individuals’ ability to carry out daily life activities such as eating, doing homework, sleeping, paying attention in class, playing, and it can lead to school absences, a poor academic performance, and even avoiding certain foods that may trigger pain. Similarly, parents of children who experienced dental pain reported increased work absences, higher costs, and a sense of guilt. Clinically, the most strongly associated condition with the presence of dental pain is advanced cavities, especially in groups with a lower socioeconomic status and limited access to dental care. Younger children with cavities tend to be more prone to higher levels of dental pain [10,11,12,13].

Toothache is a common health problem that negatively impacts an individual’s daily activities, and it is currently considered a public health issue due to the number of people it affects and the significance of its consequences. Recently, it has been observed that the prevalence of toothache throughout the lives of children and adolescents varies significantly between countries. Two systematic reviews that included both groups reported a combined overall prevalence of toothache reaching, in one, 36.2% (95% CI = 33.0–39.4) [14] and, in the other, 32.7% (95% CI = 29.6–35.9) [15].

Understanding the clinical, sociodemographic, and socioeconomic factors associated with dental pain in children will allow oral health policymakers to design more effective and equitable public policies in Mexico and similar countries. This type of evidence is essential for implementing prevention and treatment programs that are accessible and effective, especially in the pediatric/adolescent population. In Mexico, few studies have been conducted on the subject [16,17,18,19]. Therefore, the objective of this study was to evaluate the prevalence of dental pain and its association with clinical, sociodemographic, and socioeconomic risk indicators in schoolchildren aged 6 to 12 years old in four cities in Mexico.

## 2. Materials and Methods

### 2.1. Study Design, Population, and Sample

A cross-sectional analytical study was conducted with students from public primary schools in four cities in Mexico: Pachuca, Tepatitlán, Toluca, and San Luis Potosí. Part of the methodology has already been published [20,21,22]. The inclusion criteria were being an enrolled student at the selected school, being between six and twelve years old, students who gave their positive assent to participating in the study, and parents or guardians who agreed to the participation of their children in the study. The exclusion criteria were students with any intellectual disability or those with congenital defects in their dentition. Parents or guardians signed informed consent forms allowing their children to participate and completed a questionnaire.

Cluster-based, stratified sampling was used, with schools selected in proportion to their student populations. The number of schools per stratum was proportional to the total number of students, with the condition that at least two schools be visited in each city. A proportion estimation formula was applied to determine the sample size per school: n = z^2^ p(1-p)/d^2^k (1 + p(k-1)), where p = the proportion of children with at least one decayed tooth (this analysis was part of a broader project that measured multiple oral health indicators), d = the half-width of the confidence interval, p = the intra-cluster correlation coefficient, k = the number of students per school, and z = 1.96, the 97.5% quartile of a standard normal distribution. The value of p was set at 60%, leading to a final selection of 500 students. The participation rate varied across the selected cities, ranging between 76% and 95%.

### 2.2. Data Collection and Variables

Data collection was carried out in a standardized manner, and a manual was created to ensure that all data collection centers followed a single protocol in a rigorous manner. The selected schoolchildren were provided with a form requesting consent from parents or guardians for the student to be included in the study. A questionnaire was also attached to collect health, sociodemographic and socioeconomic data. To perform the clinical examination, the schoolchildren were evaluated in natural light. Each examiner used disposable gloves, a mask, a dental mirror, and a WHO-type periodontal probe.

The dependent variable was dental pain, which was dichotomized as 0 = no pain in the last 12 months and 1 = pain in the last 12 months. The independent variables were sex, age, mother’s age, and father’s age, as well as variables related to socioeconomic status such as mother’s education, father’s education, number of members who depend on the head of the family, monthly household expenditure (minimum wages in Mexico), access to medical insurance, car in the home, and two socioeconomic position (SEP) variables: the SEP of housing characteristics and SEP of household goods. Various oral health indicators were also included, such as primary and permanent decayed teeth, frequency of tooth brushing, reason for last dental visit in the last 12 months, self-reported trauma or blow to teeth or mouth, and self-reported disease in teeth and gums.

Two indicators of socioeconomic position (based on household characteristics and household items) were created using a principal component analysis approach, namely polychoric correlation analysis [23]. Tertiles were calculated for these variables, with the first tertile representing the group with the worst SEP and the last tertile the group with the best SEP.

### 2.3. Statistical Analysis

In the univariate analysis, measures of central tendency and dispersion were calculated for quantitative variables. Frequencies and percentages were calculated for qualitative variables. Binary logistic regression was used in the bivariate analysis.

In the multivariate analysis, the binary logistic regression model was used. The strength of the association between the dependent variable and the independent variables was expressed as odds ratios (ORs) with 95% confidence intervals (95% CI). The variance inflation factor (VIF) test was performed in order to analyze and, where appropriate, avoid multicollinearity between the independent variables. For the construction of the model, those variables that in the bivariate analysis showed a *p* value < 0.25 were taken into account. The overall adjustment of the model was performed with the goodness-of-fit test [24,25]. In the multivariate model, confidence intervals were calculated with robust Huber–White standard errors to obtain valid estimates, given the existence of correlation by groups (the cluster of the city variable). This was done because schoolchildren from a certain city could be more similar to each other, and therefore be more correlated than with those from other cities (difference between the clusters) [26]. Statistical analysis was performed using the Stata 14 statistical program.

### 2.4. Ethical Considerations

This project was previously evaluated and approved by the Research Ethics Committee of the Institute of Health Sciences of the Autonomous University of the State of Hidalgo (CEeI 000019-2019). It complies with the requirements of the health research laws in force in Mexico and is attached to the Helsinki standards. Parents/guardians were responsible for signing a document where they approved the use of the data for research.

## 3. Results

Table 1 presents the descriptive analysis results for the sociodemographic and socioeconomic variables. It was observed that the majority of the participants were girls (50.4%). The average age of the students was 8.92 ± 1.99 years. Regarding socioeconomic variables, the following stands out: in terms of parental education, most had more than a secondary education (mothers 54.0% and fathers 55.4%); 89.8% had some form of health insurance; 55.0% had a car in the household; the average expenditure (in terms of minimum wages) was 2.21 times; the SEP variables related to household characteristics and domestic appliances were divided into tertiles. Other sociodemographic characteristics are shown in Table 1.

Table 2 presents the results of the descriptive analysis of variables related to oral health. The average number of decayed primary teeth was 2.20 ± 2.73, while for permanent teeth, it was 0.74 ± 1.33. A frequency of tooth brushing of “twice or more per day” was reported by 52.8% of the children. Regarding dental visits, 61.2% had not seen a dentist in the previous year. Most of the students (82.0%) had no history of oral or dental trauma. Self-reported dental disease was found in 9.6% of the participants and gum disease in 3.6%. Finally, the prevalence of dental pain was 34.0%.

Table 3 shows the results of the bivariate binary logistic regression analysis for the variable dental pain in the last 12 months with each independent variable included in the study. This analysis, prior to the multivariate analysis, served to identify the independent variables with a *p* value < 0.25, and these were taken into account for the construction of the final model.

Table 4 shows the results of the bivariate binary logistic regression analysis for the variable dental pain in the last 12 months with each variable related to oral health included in the study. This analysis, prior to the multivariate analysis, served to identify the independent variables with a *p* value < 0.25 and was taken into account for the final model.

In the final multivariate model, shown in Table 5, it was observed that for each year that the individual’s age increases, the likelihood of presenting dental pain decreases (OR = 0.81, 95% CI = 0.72–0.91). Schoolchildren who had a car at home were less likely to present dental pain (OR = 0.77, 95% CI = 0.64–0.93) than students who did not have a car at home. Schoolchildren with the worst SEP (household goods) were more likely to present dental pain (OR = 2.03, 95% CI = 1.28–3.20) than schoolchildren with medium and high SESs. Children who went to a dental consultation for curative or specialized care were more likely to present dental pain (OR = 8.40, 95% CI = 3.20–21.99) than children who did not go to the dentist in the previous year. Schoolchildren whose parents/guardians had reported that their children had dental and/or gum disease were more likely (OR = 5.36, 95% CI = 3.63–8.00) to experience dental pain than those who did not report dental and/or gum disease.

## 4. Discussion

The present study aimed to determine the prevalence of dental pain and its associated factors in Mexican schoolchildren. A prevalence of 34% was observed, and some variables of a different nature associated with dental pain were found. In previous studies conducted in Mexico, dental pain prevalences of 49.9% have been reported in children aged 6 to 12 years [19] and 30.7% in children aged 2 to 12 years [17]. Similarly, but at the national level, the estimated prevalence of dental pain in Mexico was 26.9%, with the prevalence of dental pain by State ranging from 11.3% to 37.9% for elementary school students and from 14.9% to 27.1% for middle school students; the age of the study subjects was between 5 to 16 years [16]. Regarding international comparisons, the percentage observed in this study was similar to that found in India, where it was reported that 35% of subjects aged 10 to 15 experienced dental pain [27]. In another study conducted on schoolchildren aged 8 to 12 years in Nigeria, a pain prevalence of 24.9% was reported, lower than that found in this study [28]. In Latin America, the most studies have been conducted in Brazil, where prevalences range from 9.9% [29] to 11.8% [30], 21.1% [31], 21.8% [32], 22.0% [33], 25.0% [11,12], 28.7% [34], and up to 51.5% [35] in various parts of the country. On the other hand, it has been estimated that, globally, the prevalence of dental pain is 32.7%, with two out of ten children under 5 years old, four out of ten children between 6 and 12 years old, and three out of ten adolescents between 13 and 18 years old having experienced dental pain in the past [10]. Taking into account this epidemiological data, it can be revealed that dental pain is a common health problem; moreover, it is known that it can have a negative impact on a person’s daily activities and is currently considered a public health issue. This is so because of the number of people it affects, as well as the significance that its consequences may have [15,36]. The prevalence rates observed in Mexico and around the world have wide values, which may be due to different situations; for example, the studies are carried out in various contexts, where we can find different levels of development in each country and even within the same country. Likewise, the response of the health system to addressing oral health needs and the way that access to and the quality of dental care is different in each country or region of the same country can significantly influence the prevalence of dental pain; a region with limited access to dental services is likely to have a higher prevalence of untreated dental problems and consequently, greater dental pain than a region with easy access and a better quality of dental care. Moreover, the methodologies for defining pain reference periods vary across studies; some studies include two, six, and even twelve months. Likewise, the characteristics of the population studied influence the observed prevalence rates; variations in age ranges, socioeconomic level, and geographic locations play an important role in the reported percentages of dental pain between the different studies. Finally, there may be cultural biases associated with self-reported dental pain; the way pain is perceived and reported may differ between cultures, leading to inconsistencies in prevalence figures across studies. The differences between this study and other studies conducted in Mexico are, for example, in the type of sampling carried out. Previous studies conducted in Mexico generally include one population. However, in this study, schoolchildren from various places in Mexico were included (a multicenter study). Therefore, the representativeness (geographical, socioeconomic, etc.) may be better. Likewise the way of selecting the schools, where those that were selected were based on their size.

In this study, it was found that at a younger age, there was a higher likelihood of having dental pain, unlike what was observed in other studies that found a relationship with dental pain, but at an older age [17,28]. We could relate this to the fact that older children have more socialization activities, and they also incorporate rules and cultural patterns that are more related to personal care, in contrast to younger children [12]. In younger children living in middle-income countries, such as Mexico, there are factors that contribute to the presence and development of cavities, such as the lack of proper dental hygiene, a diet rich in sugars from frequently consuming snacks or sugary drinks between meals and even before going to bed, and the lack of oral health services and additional preventive measures. All these factors cause a large number of cavities in both primary and permanent dentition, which can lead to the onset of dental pain [37,38,39,40]. Additionally, in the present study, more children with primary dentition were observed, where the disease is likely to be more concentrated.

The worst socioeconomic characteristics are important factors that can contribute to the onset of oral diseases in children and adolescents. In this study, several indicators of socioeconomic status remained in the final model, significantly associated with a higher likelihood of experiencing dental pain, similar to what was found in other studies [17,19]. Health inequalities are well established, characterized by social gradients, where groups of a lower socioeconomic position have a higher risk of oral disease and therefore dental pain [41]. This study also used socioeconomic indicators that differ from other studies, two of which remained in the final model (household car ownership and household goods). These could be integrated into studies in different settings. The study does not reveal radically new results, but it strengthens the previous evidence, provides data on the prevalence of dental pain in Mexico, and includes a broader analysis of risk factors. This information could be of interest to general and pediatric dentists who care for dental pain in children and can help them plan preventive and therapeutic interventions based on greater scientific evidence of what complaints need to be addressed, particularly in the context of Mexico.

The data obtained in the present study showed an association between dental pain and children who attended dental consultations for curative or specialized care. Similarly, there is an association between self-reported dental and/or gum disease and pain. It is necessary to identify that differences in perceptions of oral health hinder comparisons of self-reported oral health in countries with cultures, traditions, and dental care that are considerably different [31]. These results are similar to those previously found in Mexico [17]. This can be explained by the fact that Mexico is a middle-income country where the majority of the population, especially children and adolescents, has limited access to preventive and curative dental health services [27]. Moreover, this happens where there is a high prevalence of caries, and many of these lesions do not receive timely dental treatment [42]. Mexico has one of the highest rates of dental caries in the world. Some national studies have found that around 50% of students aged 5 to 16 have or have reported having cavities [43]. It is also estimated that between 70% and 85% of 12-year-old children have dental caries in their permanent dentition, and 50% of 6-year-old children have caries in their primary dentition [44]. According to the data from a systematic review, the global prevalence of dental caries in primary teeth is 46.2% and in permanent teeth is 53.8% [45]. In Latin America, dental caries has a prevalence of 56% in primary dentition and 58% in permanent dentition [41]. To prevent dental pain in children and thus achieve a better quality of life, it is necessary to strengthen preventive and therapeutic dental programs and services for the child population [46]. According to previous studies, it was revealed that children with dental pain sought dental care for dental pain more than for preventive care [11,31]. The association between dental pain and schoolchildren who had visited the dentist is concerning, as a dental consultation should encourage greater and better care, as well as providing treatment for dental problems, resulting in relief from pain. Therefore, it is possible that the dental services used did not resolve the pain issues [31].

The present study could have an impact on oral health policies in Mexico. The high prevalence of dental pain (1 in 3 children) found in the study highlights the need to implement more effective prevention programs in the country. The association of dental pain with low socioeconomic levels and lack of access to specialized dental care indicates that interventions should focus on vulnerable populations. Educational programs aimed at children, parents, and educators on the importance of oral hygiene, a healthy diet, and caries prevention should be tailored to the cultural and economic context of each community. In addition, access to oral health services should be affordable and of good quality, especially in areas with low economic resources, and it should be implemented using various strategies, such as mobile dental care or through cost subsidies. These government oral health programs should be strengthened with a larger budget, trained personnel, and more effective strategies for the prevention and control of dental caries, which is the main cause of dental pain. On the other hand, the results of this study emphasize the importance of monitoring the prevalence of dental pain in Mexican children. Thus, the creation of an epidemiological surveillance system is proposed to identify trends, evaluate the impact of interventions, and adjust strategies as necessary. In summary, the public health implications of this study require a multisectoral response to address the underlying causes of dental pain and promote the oral health of Mexican children. Likewise, collaboration between government institutions, health professionals, education, and the community is essential to achieve a positive and lasting impact.

The present study has limitations that must be taken into account for its correct interpretation. One of them is inherent to its cross-sectional design, as measuring both the cause and the effect simultaneously introduces the bias known as temporal ambiguity, where causal relationships cannot be established. It can only identify associations, but it cannot determine if the risk factors are the direct cause of dental pain. The dependent variable is self-reported dental pain, which may be subject to recall bias and subjectivity, although self-reporting is a common and valid measure in epidemiological studies. On the other hand, the results may not be generalizable to other regions of Mexico or to other countries with different sociodemographic, cultural, or economic characteristics. The sample was taken from public schools, which may bias the results towards a particular group of children, excluding those who attend private schools, who might have a different access to dental care and present different risk profiles.

## 5. Conclusions

Dental pain is a significant health problem in schoolchildren aged 6 to 12 years in Mexico, with a prevalence of 34% in this study. Sociodemographic and socioeconomic factors, as well as clinical indicators, play an important role in the experience of dental pain. In this study, socioeconomic variables were found to be associated with dental pain, which suggests the existence of socioeconomic inequalities in this oral health indicator.

Strengthening prevention programs and improving access to dental services in Mexico, particularly in socioeconomically vulnerable areas, is essential. Additionally, the importance of prioritizing education and the promotion of oral health from an early age is emphasized to reduce the prevalence of cavities and other dental diseases that lead to pain.

## Figures and Tables

**Table 1:** Descriptive analysis of the independent sociodemographic and socioeconomic variables included in the study.

Variable	Mean	SD
Child’s age	8.92	1.99
Maternal age	34.55	6.08
Paternal age	36.98	6.63
Members who depend on the head of the family	3.27	0.98
Monthly household expenditure	2.21	0.96
	Frequency	Percentage
Sex		
Boys	248	49.6
Girls	252	50.4
Mother’s education		
Up to secondary school	230	46.0
More than secondary school	270	54.0
Father’s education		
Up to secondary school	223	44.6
More than secondary school	277	55.4
Health insurance		
With insurance	449	89.8
Without insurance	51	10.2
Car at home		
No	225	45.0
Yes	275	55.0
SEP (housing characteristics)		
1st tertile (worst SEP)	192	38.4
2nd tertile	148	29.6
3rd tertile (best SEP)	160	32.0
SEP (household goods)		
1st tertile (worst SEP)	167	33.4
2nd tertile	186	37.2
3rd tertile (best SEP)	147	29.4

**Table 2:** Descriptive analysis of the variables related to oral health included in the study.

Variable	Mean	SD
Primary decayed teeth	2.20	2.73
Permanent decayed teeth	0.74	1.33
	Frequency	Percentage
Tooth brushing frequency		
Less than twice a day	236	47.2
Two or more times a day	264	52.8
Reason for last dental visit		
Never/not in the last year	306	61.2
Curative or specialized	94	18.8
Preventive care	100	20.0
Trauma or blow to teeth or mouth		
No	410	82.0
Yes	90	18.0
Does your child have any dental disease?		
No	452	90.4
Yes	48	9.6
Does your child have any gum disease?		
No	482	96.4
Yes	18	3.6
Toothache		
No	330	66.0
Yes	170	34.0

**Table 3:** Bivariate binary logistic regression analysis for dental pain in the last 12 months and the sociodemographic and socioeconomic variables of the study.

Variable	OR (95% CI)	*p* Value
Child’s age	0.84 (0.78–0.90)	<0.001
Maternal age	0.99 (0.95–1.02)	0.642
Paternal age	1.01 (0.97–1.04)	0.513
Members who depend on the head of the family	1.14 (0.94–1.38)	0.178
Monthly household expenditure	0.89 (0.79–1.01)	0.098
Sex		
Boys	1 *	
Girls	0.78 (0.41–1.48)	0.460
Mother’s education		
Up to secondary school	1 *	
More than secondary school	1.06 (0.53–2.14)	0.856
Father’s education		
Up to secondary school	1 *	
More than secondary school	1.16 (0.85–1.57)	0.331
Health insurance		
With insurance	1 *	
Without insurance	1.28 (0.52–3.15)	0.583
Car at home		
No	1 *	
Yes	0.63 (0.54–0.74)	<0.001
SEP (housing characteristics)		
1st tertile (worst SEP)	1 *	
2nd tertile	0.61 (0.31–1.18)	0.144
3rd tertile (best SEP)	0.34 (0.07–1.55)	0.166
SEP (household goods)		
1st tertile (worst SEP)	1 *	
2nd tertile	0.38 (0.27–0.53)	<0.001
3rd tertile (best SEP)	0.24 (0.11–0.49)	<0.001

* Reference category. Note: Confidence intervals with robust standard Huber–White errors were calculated.

**Table 4:** Bivariate binary logistic regression analysis for dental pain in the last 12 months and the oral health-related variables of the study.

Variable	OR (95% CI)	*p* Value
Primary decayed teeth	1.09 (0.94–1.26)	0.218
Permanent decayed teeth	1.06 (0.90–1.26)	0.444
Total number of decayed teeth	1.13 (0.99–1.28)	0.065
Tooth brushing frequency		
Less than twice a day	1 *	
Two or more times a day	0.33 (0.07–1.47)	0.149
Reason for last dental visit		
Never/not in the last year	1 *	
Curative or specialized	8.50 (3.03–23.83)	<0.001
Preventive care	1.39 (0.56–3.40)	0.468
Trauma or blow to teeth or mouth		
No	1 *	
Yes	1.22 (0.44–3.37)	0.697
Does your child have any dental disease?		
No	1 *	
Yes	6.32 (3.42–11.66)	<0.001
Does your child have any gum disease?		
No	1 *	
Yes	17.03 (4.10–70.72)	<0.001

* Reference category. Note: Confidence intervals with robust standard Huber–White errors were calculated.

**Table 5:** Multivariate binary logistic regression model for the prevalence of dental pain.

Variable	Odds Ratio	(95% CI)	*p* Value
Child’s age	0.81	0.72–0.91	<0.001
Car at home			
No	1 *		
Yes	0.77	0.64–0.93	0.008
SEP (household goods)			
1st tertile (worst SEP)	2.03	1.28–3.20	0.002
2nd and 3rd tertile (best SEP)	1 *		
Reason for last dental visit			
Never/not in the last year	1 *		
Curative or specialized	8.40	3.20–21.99	<0.001
Preventive care	1.47	0.66–3.31	0.342
Self-reporting of dental and/or gum disease			
No	1 *		
Yes	5.39	3.63–8.00	<0.0001

* Reference category. Note: Estimates adjusted for variables contained in table in addition to sex. Confidence intervals with robust standard Huber–White errors were calculated. Goodness-of-fit test: Hosmer–Lemeshow Chi-square (8) = 11.28, *p* value = 0.1862.

## Data Availability

The datasets generated and/or analyzed during the current study are available from the corresponding author upon reasonable request.
